# Rocking the Cradle: Phthalate Exposure in NICU Infants

**Published:** 2005-09

**Authors:** Julia R. Barrett

Animal studies have linked di(2-ethylhexyl) phthalate (DEHP) with reproductive and developmental toxicity, and have demonstrated an especially pronounced effect on testicular development when administered postnatally. Previous research has shown that newborns treated at neonatal intensive care units (NICUs) may receive doses of DEHP at 2–3 times the average daily adult exposure, and that these infants have relatively high urinary levels of the DEHP metabolite mono(2-ethylhexyl) phthalate (MEHP). Now researchers, using urinary MEHP as a biomarker of DEHP exposure, demonstrate for the first time that the more DEHP-containing devices are used in treating an infant, the more DEHP makes its way into the infant’s body **[*EHP* 113:1222–1225]**.

Human DEHP exposure is widespread but generally much lower than the levels causing harm in animal studies. However, certain circumstances such as intensive medical treatment can result in higher-than-average exposure, which may be a particular risk for newborn males. DEHP is added to polyvinyl chloride (PVC) plastics for use in medical equipment including IV bags, blood bags, and various types of tubing, as well as many industrial and consumer PVC products. DEHP does not chemically link to PVC and leaches into fluids (such as blood and saline solution) that contact the plastic. The amount of leaching depends upon factors such as type of fluid, length of storage, and temperature.

The study involved 54 newborn girls and boys receiving treatment at two Boston-area NICUs between 1 March and 30 April 2003. The infants had been admitted for various reasons, and treatment included procedures such as mechanical ventilation, enteral feedings, and cardiac catheterization.

Prior to visiting the NICUs, the researchers defined low, medium, and high DEHP exposure categories based upon typical NICU procedures and equipment. Infants whose treatment consisted primarily of bottle and/or gavage feedings composed the low-exposure group. Infants in the medium-exposure group received more invasive therapies involving equipment such as an indwelling gavage tube or umbilical vein catheter. High-exposure infants experienced multiple and simultaneous invasive treatments, including endotracheal intubation and continuous umbilical vein catheterization.

One researcher visited the NICUs and observed each infant for 3–12 hours over the course of 1–3 days (more than one infant was observed at a time). During the observational visits, the researcher noted the equipment being used for each infant, then assigned the infant to an exposure group accordingly. At the end of each visit, urine samples were collected for MEHP measurement.

The researchers detected 10 phthalate metabolites in the samples, including 3 associated with DEHP, but focused on MEHP for data analysis since this metabolite is well studied and a proven biomarker of DEHP exposure. MEHP levels ranged from less than the level of detection to 758 nanograms per milliliter and did not vary substantially between multiple individual samples.

Between the two NICUs there were 13, 24, and 17 infants in the low-, medium-, and high-exposure groups, respectively. The researchers found that infants in the high-exposure group had MEHP levels five times higher than those in the low-exposure group. MEHP levels for medium-exposure infants were twice those of the low-exposure group.

The researchers indicate that the MEHP levels seen in this study are similar to those previously reported for NICU infants and higher than those reported for older children; no data are available for infants who did not need NICU care. The relevance of these exposures to health effects is unknown, and the researchers urge larger, more comprehensive studies with follow-up to determine consequences of DEHP exposure related to NICU treatment.

## Figures and Tables

**Figure:**
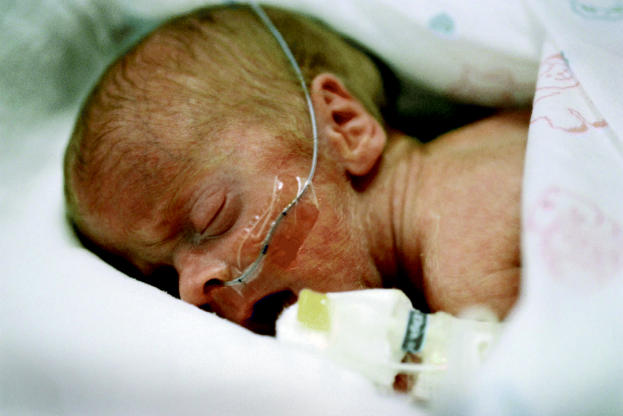
Double jeopardy? Babies in neonatal intensive care units, already a high-risk group, are likely to have greater exposure to potentially harmful phthalates than other children.

